# Engineering SIV-resistant macaque hematopoietic stem cells and CD4+ T cells with CCR5-specific zinc-finger nucleases

**DOI:** 10.1186/1742-4690-9-S2-P226

**Published:** 2012-09-13

**Authors:** G Bonello, P Lentz, M Salas, M Cepeda, R White, M Gauduin

**Affiliations:** 1 Texas Biomedical Research Institute, San Antonio, TX, USA

## Background

CCR5 is the major HIV co-receptor, and individuals homozygous for a 32-bp deletion in CCR5 are resistant to infection by CCR5-tropic HIV-1. Zinc finger nuclease (ZFN) technology is a class of engineered DNA-binding proteins facilitating targeted genome editing by binding to a user-specific locus and causing a double-strand break in the region of interest. As a result, the gene of interest targeted by ZFN cleavage is disrupted. We investigated the ability of a Ccr5 gene-specific ZFN to establish SIV-resistant CD4+ T cells and hematopoietic progenitor cells isolated from macaques.

## Methods

Bone marrow and blood samples were obtained from ten naïve uninfected rhesus macaques. Immunomagnetic enrichments were performed to isolate hematopoietic stem/progenitor cells and CD4+ T cells using magnetic-beads. Purified cells were nucleofected with RNAs encoding for a Ccr5-specific ZFNs.

## Results

We successfully engineered CCR5-modified macaque CD4+ T lymphocytes that were resistant to in vitro infection with SIVmac239, SIVmac251, and CCR5-tropism SIVagm. CD4+ T lymphocytes that incorporated RNAs encoding for the Ccr5-specific set of ZFNs marked resistance to SIV infection as showed by lack of p27 expression in culture supernatants. Infection of non-modified CD4+ T lymphocytes led to significant p27 production from day 5 post-infection with a pick at day 7 post-infection. We developed and optimized the conditions for proper isolation, expansion and in vitro differentiation of macaque hematopoietic stem cells to be used for the generation of SIV-resistant macaque hematopoietic stem cells using our ZFNs. We successfully engineered CD34+ hematopoietic stem cells using nucleofection of RNAs encoding for a Ccr5-specific ZFNs.

## Conclusion

We demonstrated the feasibility of using ZFN technology to establish CD4+ and hematopoietic stem cells resistant to SIV infection in macaque model. The generation of a nonhuman primate model using this modern molecular-based strategy might significantly help in the design of new therapies to prevent viral infection in human.

